# Conspecific Leaf Litter-Mediated Effect of Conspecific Adult Neighborhood on Early-Stage Seedling Survival in A Subtropical Forest

**DOI:** 10.1038/srep37830

**Published:** 2016-11-25

**Authors:** Heming Liu, Guochun Shen, Zunping Ma, Qingsong Yang, Jianyang Xia, Xiaofeng Fang, Xihua Wang

**Affiliations:** 1 School of Ecological and Environmental Sciences, East China Normal University, Shanghai 200241, China; 2 Tiantong National Forest Ecosystem Observation and Research Station, Ningbo, Zhejiang 315114, China

## Abstract

Conspecific adults have strong negative effect on the survival of nearby early-stage seedlings and thus can promote species coexistence by providing space for the regeneration of heterospecifics. The leaf litter fall from the conspecific adults, and it could mediate this conspecific negative adult effect. However, field evidence for such effect of conspecific leaf litter remains absent. In this study, we used generalized linear mixed models to assess the effects of conspecific leaf litter on the early-stage seedling survival of four dominant species (*Machilus leptophylla*, *Litsea elongate*, *Acer pubinerve* and *Distylium myricoides*) in early-stage seedlings in a subtropical evergreen broad-leaved forest in eastern China. Our results consistently showed that the conspecific leaf litter of three species negatively affected the seedling survival. Meanwhile, the traditional conspecific adult neighborhood indices failed to detect this negative conspecific adult effect. Our study revealed that the accumulation of conspecific leaf litter around adults can largely reduce the survival rate of nearby seedlings. Ignoring it could result in underestimation of the importance of negative density dependence and negative species interactions in the natural forest communities.

Negative conspecific interaction is of important significance for understanding coexistence of species in many communities. It can promote stable coexistence of species when its strength is stronger than the negative interspecific interactions (e.g. interspecific competition)^1^,^2^. This interaction becomes more intense among individuals with high density (e.g. negative density dependent effect)^3^, thus it is commonly expected that conspecific adult trees have widespread and strong negative effect on seedlings surrounding them. This expectation has been confirmed by many common garden experiments using various populations^4^,^5^,^6^,^7^, but is not widely supported in natural forest communities^8^,^9^. In general, the percentage of plant species with significant effect of conspecific adult neighbors on seedlings is only around 36%, even if the effect of other confounding processes like environmental heterogeneity has been rigorously controlled^8^. Thus, there is a high chance that the effect of conspecific adults on seedlings has been underestimated.

The possible underestimation might rise from our limited knowledge about how adults negatively affect their conspecific seedlings in natural forest communities. Although it is commonly accepted that host-specific natural enemies, such as fungal pathogens and herbivores, are the main cause of massive seedling mortality around conspecific adults^10^,^11^, we know few about how these natural enemies accumulate around the adults. For example, where does the increasing host-specific soil biota near the parent trees come from? Is it simply mediated and transported by the roots of adults in the soil? Are there any other important ways to carry host-specific enemies from adults to the nearby seedlings? These uncertainties in turn limit our ability to accurately quantify and estimate the effect of adults on their conspecific seedlings in natural communities.

Here, we hypothesized that conspecific leaf litter could be another important alternative media for the negative effect of conspecific adult neighborhood on seedling survival. Leaves, as the main part of above-ground adult trees, are often attacked by foliar fungal pathogens (intercept most of rain or wind-borne spores)^12^,^13^,^14^. They will finally fall to the nearby ground where most conspecific seedlings distributed. Therefore, the leaf litter may transfer fungal pathogens on adults to seedlings^15^. García-Guzmán and Benítez-Malvido (2003) have found the seedlings survival rate of *Nectandra ambigens* was reduced by the leaf-fungal pathogen when they added the leaf litter most from the parent tree. Thus they inferred that leaf-fungal pathogen brought by conspecific leaf litter is the most important reason of the reduced performance of their seedlings. Up to now, there were few studies examined the potential effect of leaf litter-mediated fungal pathogens on seedling establishment in natural forest communities^16^.

Most conspecific leaf litter covering the seedlings comes from the adult neighbors, so it is commonly assumed that the patterns of conspecific leaf litter and conspecific adult neighborhood are highly correlated. Then commonly applied methods (e.g. neighborhood indices), which simply assume that the negative effect of adult decreases with the distance from adult to the seedlings and is isotropic in each direction^17^,^18^,^19^,^20^,^21^,^22^, would pick up the effect of conspecific leaf litter on the seedling survival. However, when the correlation is weak, the negative effect of conspecific leaf litter on seedlings will not be as simple as described in commonly applied methods. In the natural communities, the distribution of leaf litter is generally decreased with the distance from the parent trees, but is largely influenced by wind speed, direction and forest vertical structure^23^. These influences will be much stronger for species with light and large leaves^24^, and will be different in strength on different topological ground. Because the distribution of leaf litter probably largely biased from isotropic shape, the negative effect of adult on seedlings mediated by leaf litter might be anisotropic from the parent tree^25^,^26^,^27^. This ignored fact of the complex spatial intensity of adult effect on conspecific seedlings proposed a unique challenge to robustly evaluate the importance of conspecific adult effect on seedlings by conventional neighborhood indices in natural forest communities.

To overcome this challenge and present the first field examination of whether conspecific leaf litter is an important way to mediate the negative adult effect on conspecific seedlings, observed leaf litter on 561 seedling plots was used to explain the early-stage survival of the seedlings in a subtropical forest. Specifically, we built two types of generalized linear mixed models to address the following questions: (1) Do the amount of conspecific leaf litter significantly affect the early-stage seedling survival of the chosen tree species? (2) If so, can we found this indirect effect by the conventional conspecific adult neighborhood indices? Comparing results of above two analyses finally could reveal whether the negative effect of conspecific adults on seedlings is indeed underestimated in our natural forest community.

## Results

### The effect of conspecific adult neighbors through leaf litter

**Figure 1:**
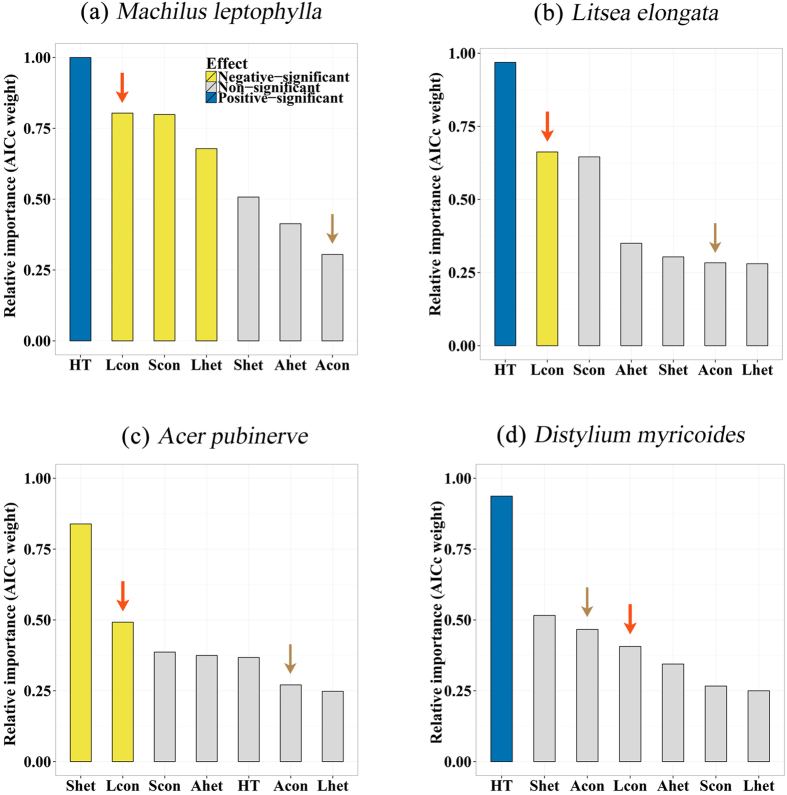
The relative importance of potential factors (including conspecific and heterospecific leaf litter) influencing on early-stage seedlings survival of four species. The potential factors were the log-transformed initial height (HT) of seedlings, the amount of conspecific (Lcon) and heterospecific (Lhet) leaf litter, conspecific adult neighborhood indices (Acon) and heterspecifics (Ahet), the density of conspecific (Scon), and heterospecific (Shet) seedling neighbors. Blue bins indicate significant, positive effects of the variables on seedling survival (The model-average estimator 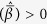); while yellow bins indicate significant, negative effects (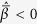); gray bars indicate non-significant effects (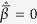). The red and brown arrows highlight the variable of conspecific leaf litter and conspecific adult neighborhood indices respectively.

The leaf litter from adult neighbors generally had a strong negative effect on the survival of conspecific seedlings (Fig. 1). Except for the *Distylium myricoides* with the least abundance of newly germinated seedlings among the four examined species, the leaf litter of other three species can significantly reduce the survival rate of their conspecific seedlings. Moreover, the strength of this inhibition from conspecific leaf litter was stronger than the effect from the conspecific seedling neighbors, and became the second strongest factor among seven potential variables in three of four examined species (ranks in Fig. 1). In addition, the negative effect of leaf litter on seedling survival seems to have a moderate species specific trend, because the effect of conspecific leaf litter on seedling survival was more important than heterospecific leaf litter in all examined species.

### The effect of conspecific adult neighbors through neighborhood indices

**Figure 2:**
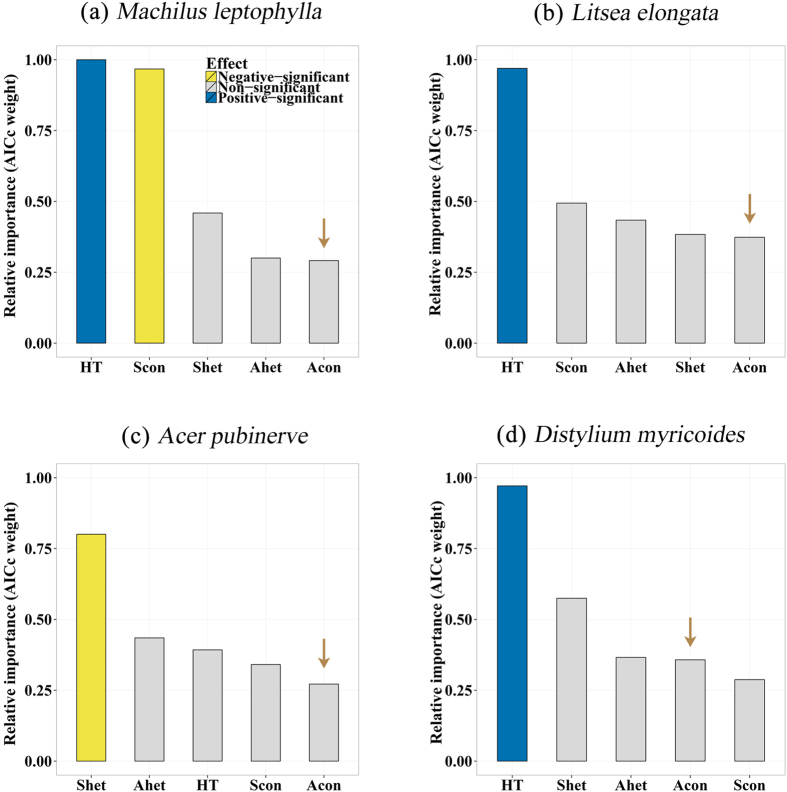
The relative importance of potential factors (excluding conspecific and heterospecific leaf litter) influencing on early-stage seedlings survival of four species. The potential factors were the log-transformed initial height (HT) of seedlings, conspecific adult neighborhood indices (Acon) and heterospecific (Ahet), the density of conspecific (Scon) and heterospecific (Shet) seedling neighbors. Blue bins indicate significant, positive effects of the variables on seedling survival (The model-average estimator 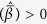); While yellow bins indicate significant, negative effects (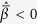); Gray bins indicate non-significant effects (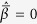). The brown arrows highlight the variable of conspecific adult neighborhood indices.

The conspecific adult neighborhood indices, however, did not have significant, negative effect on seedling survival of the whole chosen species. This result is robust with respect to either exclude or include the variable of conspecific leaf litter (Figs 1 and 2). Meanwhile, the relative importance of conspecific adult neighborhood indices were the lowest among four potential variables of biotic neighbors in three of four examined species (Fig. 1a,b,c), even though the leaf litter variable was removed in the analyses (Fig. 2a,b,c). Therefore, the effect of conspecific leaf litter on seedling survival did not largely included in the effect of conspecific adult neighborhood indices. On the other hand, the conspecific and heterospecific seedling neighborhood indices (density of conspecific and heterospecific seedlings in the same seedling plot as the focal seedling) had significant, negative effects on seedling survival of *Machilus leptophylla* (Figs 1a and 2a) and *Acer pubinerv* (Figs 1c and 2c), respectively.

### Correlation between conspecific leaf litter and adult neighborhood indices

**Figure 3:**
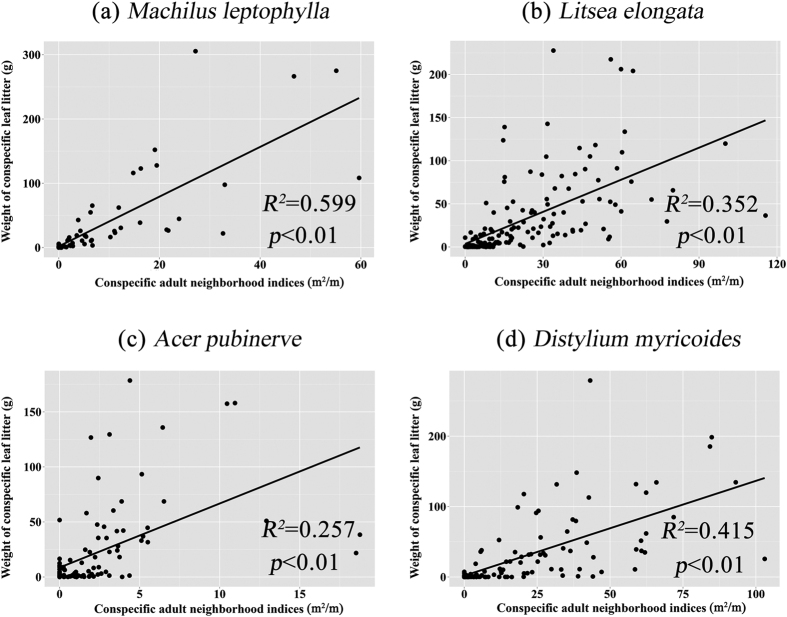
Relationship between sum of the weight of conspecific leaf litter from 2011 to 2013 in the litter trap and the conspecific adult neighborhood indices, i.e, sum of the basal area (m^2^) divided by distance (m) from the focal litter trap within 10 m. *R*^2^ is the adjusted R-squared.

There was substantial difference between the spatial variation of the conspecific leaf litter and the conspecific adult neighborhood indices (Fig. 3). The difference was mostly significant for the *Acer pubinerv* (Fig. 3c). In addition, the potential explanatory variables of conspecific leaf litter and conspecific adult neighborhood indices did not cooccur in the optimal model groups of *Machilus leptophylla*, *Litsea elongata* and *Acer pubinerv* as the independent variables (Table S1 in appendix). Therefore, the multiple-collinearity of these two potential explanatory variables did not influence the effect of conspecific leaf litter on seedling survival of these three species.

## Discussion

Conspecific leaf litter was almost completely ignored in most of previous field studies testing the negative conspecific density dependent effect, thus its potential importance in forest community was still largely unexplored. Our study filled this gap and provided the first empirical support on the contribution of conspecific leaf litter to tree seedling survival. Several of our results consistently indicated that the amount of conspecific leaf litter had strong negative effect on early-stage seedling survival of three species (*Machilus leptophylla*, *Litsea elongata* and *Acer pubinerve*). Because 91.5% of 2458 newly germinated seedlings during the study period belonged to these three species, it is highly probable that the conspecific leaf litter widely mediate the effect of conspecific adult neighborhood on the early-stage seedling survival in our Tiantong plot. The conspecific leaf litter could be an another important media for the negative conspecific density dependence.

Indirect evidences from other studies suggest that the conspecific leaf litter can inhibit the conspecific seedling survival through different ways in multiple species. The diseases of tree species would be caused by species- or genus-specific foliar pathogens (*Machilus leptophylla - Pestalotia neglecta*, *Litsea - Uncinula SP*, *Acer - Phyllosticta aceris & Uncinula nankinsensis*)^28^,^29^,^30^,^31^. The conspecific leaf litter may play as the carrier of leaf-fungal pathogens which can inhibit the seedling survival of the three tested species. In addition, the leaf of *Acer pubinerve* (*Aceraceae*) contains much cis-3-hexenyl acetat^32^ which are herbivore-induced plant volatiles^33^. These volatiles would attract natural enemies of the herbivores during the leaf litter decomposition^34^. Therefore, the seedling of *Acer pubinerve* would be increasingly fed by natural enemies of the herbivores in the accumulation of conspecific leaf litter.

Except for the biological attraction effect of pathogens and herbivores, conspecific leaf litter can also increase the conspecific seedling mortality through chemical effect^35^. The leaves of *Machilus* and *Litsea* contain much alkaloid^36^,^37^ which is an important allelochemical^38^. The leaf litter of *Machilus leptophylla* (Lauraceae) and *Litsea elongata* (Lauraceae) may inhibit the conspecific seedling survival through the autotoxicity. However, it is hard to answer in current study that whether conspecific leaf litter inhibits the seedling survival through the biological process in bitrophic level or the chemical process in unitrophic level. Manipulation experiments are needed to fully understand how conspecific leaf litter affects seedling performance.

The conspecific leaf litter-mediated effect of conspecific adult neighbors on early-stage seedling survival should be detected by the conspecific adult neighborhood indices directly. However, the commonly used conspecific adult neighborhood indices did not have significant effect on seedling survival in our studies (Fig. 2). One possible reason is that this commonly used conspecific adult neighborhood indices fail to capture this indirect negative conspecific adult effect on seedlings, particular in an environment with rough topography and strong wind disturbance. The weak relationship in our study between these conspecific adult neighborhood indices and the amount of conspecific leaf litter partly supports our speculation.

This conspecific adult neighborhood indices was designed to evaluate conspecific neighborhood effect that is homogeneous from the conspecific adult neighbor circumference^18^,^39^. However, the leaf dispersal would be inhomogeneous in the circumference of these species in the Tiantong plot. This inhomogeneous pattern of leaf dispersal could be caused by many reasons. The major reason would be the leaf shapes of the chosen species were broad leaf and our study site was influenced by subtropical monsoon^40^,^41^. The wind directions would affect broad leaf dispersal^23^,^27^. Especially, the most wind-influenced maple leaves of *Acer pubinerv* (others have leathery leaves)^42^,^43^,^44^ show the largest difference between the distribution of conspecific leaf litter and conspecific adult neighborhood indices (Fig. 3). Meanwhile, the topography of our plot was rough^41^. The amount of leaf litter would vary in the different microtopography. Because of these difference between the conspecific adult neighborhood indices and distribution of conspecific leaf litter, it is not supprise to find that the conspecific adult neighborhood indices can not detected this conspecific leaf litter- mediated effect of conspecific adult neighbors on seedling survival in the Tiantong plot.

As a consequence, the above weakness of conspecific adult neighborhood indices could result in large underestimation of the importance of negative density dependence in natural forest communities. Considering the importance of conspecific leaf litter on seedling survival offers one possible solution. Majority of previous experiments only tested the soil-mediated effect of adult neighbors on seedling survival^6^,^7^,^45^,^46^,^47^, and rarely considered the potential impact of conspecific leaves on seedling demographic rates. In the future, we should design a manipulation experiment to make up this gap. In addition, the habitat preference could be an important mechanism for influencing the early-stage seedling survival^48^,^49^,^50^. In two of four species, the environmental effects explained total variance of seedling survival more than these biotic factors (Table S3 in appendix). This phenomenon indicated that the effect of conspecific leaf litter on seedling survival would be overlaid with the environmental effects in some species (Figure S1 in appendix).

In summary, our study revealed that the accumulation of conspecific leaf litter around adults can reduce the survival rate of nearby seedlings, and provide a certain advantage around adults for the generation of other species. The conspecific leaf litter might be an important media for the negative effect of conspecific adult neighborhood on early-stage seedling survival in Tiantong plot. Ignoring this negative effect of conspecific leaf litter could result in large underestimation of the importance of negative density dependence and negative species interactions in the natural forest communities.

## Methods

### Data collection

**Figure 4:**
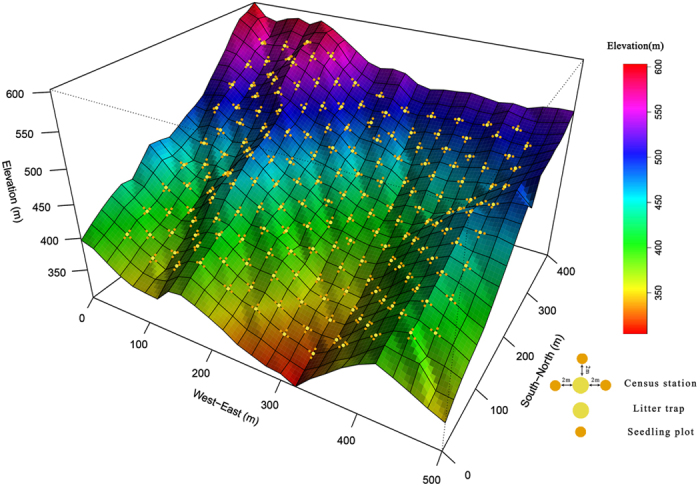
Distribution of seedling census stations and the litter traps in the Tiantong plot. Yellow points and orange points represented 0.5 m^2^ of litter traps and 1 m^2^ seedling plots, respectively.

We conducted the study in a 20-ha (500 × 400 m) forest dynamics plot in a subtropical evergreen broad-leaved forest in the Tiantong Forest Park (29°48′N, 121°47′E) (hereafter called Tiantong plot), eastern China^41^. This region has a subtropical monsoon climate and receives 1374.7 mm mean annual rainfall. The annual average temperature is 16.2 °C. The minimum and maximum monthly mean temperatures are 4.2 °C in January and 28.1 °C in July, respectively. The soil texture is mainly red-yellow soil and pH value of the soil is from 4.5 to 5.0^40^. The topography of the Tiantong plot is rough^41^, with elevation ranges from 304.26 to 602.89 m. There are two large ridges throughout the plot from south to north (Fig. 4). All free standing trees (DBH ≥ 1 cm) in the Tiantong plot have been mapped, identified to species and measured in 2010. The dominant families of trees in the Tiantong plot are Theaceae, Lauraceae and Fagaceae which are widely distributed in the subtropical evergreen broad-leaved forest^41^.

To monitor seedling and leaf litter dynamics in the Tiantong plot, 187 census stations were established in two opposite corners of each 20 × 20 m plots in the Tiantong plot (Fig. 4). The census stations were at least 40 m far away from the border of the Tiantong plot to avoid edge effects. Each station consisted of a 0.5 m^2^ litter trap in the center and three 1-m^2^ seedling plots surrounded the litter trap. The seedling plots were located 2 m away from the litter trap in three random directions (Fig. 4). In each seedling plot, the newly germinated seedlings were tagged, measured and identified to species in October 2011, April 2012 and October 2012. We analyzed the first-year survival of these newly germinated seedlings. The period of seedling survey for these newly germinated seedlings was from October 2011 to October 2012, from April 2012 to April 2013 and from October 2012 to October 2013 respectively. In addition, other woody plants with DBH ≤ 1 cm in the seedling plot have been tagged, measured and identified to species. Leaf litter in each litter trap was collected every half month from October 2011 to October 2013. After the collection, the leaf litter in each litter trap was identified and grouped into different species immediately in the lab, and dried under 75 °C in an electronic oven for 72 hours. Dry weight of leaf litter for each species in each litter trap was recorded.

A total of 2458 newly germinated seedlings were observed in the 187 seedling plots in the three censuses. Most of them (94.39%) belong to *Machilus leptophylla* (Lauraceae), *Litsea elongata* (Lauraceae), *Acer pubinerve* (Aceraceae) and *Distylium myricoides* (Hamamelidaceae), with 2051, 116, 82, 71 individuals respectively. Other species only contributed around 5.6% of total newly germinated seedlings, thus were not considered in the analyses of this study to avoid problems of small sample size.

### Data analyses

Generalized linear mixed-effects models (GLMMs) with binomial errors were used to examine how the potential factors influenced early-stage seedling survival. The model with the random effects can be specified as:


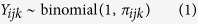



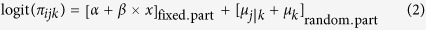


Where *Y*_*ijk*_ is 1 if newly germinated seedling *i* in the seedling plot *j* of station *k* alive in the first year and 0 otherwise, *π*_*ijk*_ is the survival probability of the focal seedling; The random part of equation (2) has two levels, first level is seedling plot *j* belonging census station *k* (*μ*_*j|k*_) and seconds is census station *k* (*μ*_*k*_)^51^,^52^. In the fixed part, *α* and *β* refer to an intercept and a vector of coefficients of explanatory variables *x*, respectively.

The potential explanatory variables in the first type of GLMMs were biotic neighbors (see details in the following paragraph), initial height and leaf litter (divided into conspecific and heterospecific groups). Because, the previous studies showed that the biotic neighbors and initial height were the major factors controlling the early-stage seedling survival^18^,^21^,^22^,^53^. Meanwhile our study is to examine whether the amount of conspecific leaf litter significantly affect the early-stage seedling survival.

The potential explanatory variable of conspecific leaf litter was defined as the conspecific leaf litter in the litter trap within the same census station as the focal seedling, and calculated by the total weight of the first year from the focal seedling germinated. The heterospecific leaf litter is total weight of the other species’ leaf litter. The potential explanatory variables of biotic neighbors were divided into conspecific and heterospecific seedling and adult neighbors. The seedling neighbors were measured by the density of conspecific or heterospecifc seedlings (DBH ≤ 1 cm) in the same seedling plot as the focal seedling^18^,^20^,^22^,^54^. The adult neighbors were measured by the adult neighborhood indices. The effects of adult neighbors tend to weaker with the distance from the adults to focal seedlings, but that effects increased with total basal area of these adults^18^. Therefore adult neighborhood indices were defined as the total of the basal area of conspecific or heterospecific adults (DBH ≥ 1 cm) within 10 m^18^,^22^,^39^ divided by the distance that tree from the center of the focal seedling plot. If the conspecific or heterospecific adults are in the focal seedling plot, the value of distance will be short abnormally. This extremely short distance will cause the adult neighborhood indices large abnormally. Therefore we assumed that trees (in the focal seedling plot) in the edge of the focal seedling plot (1 m × 1 m) and adjusted their distances to the focal seedling plot into 0.5 m.

If the conspecific leaf litter affects early-stage seedling survival negatively, there will be a negative conspecific interaction between the adults and seedlings. To detect this negative conspecific interaction through the common methods, we built the second type of GLMMs by removing potential explanatory variables of leaf litter from the first type of GLMMs. The reason was the effect of leaf litter would disturb the effect of biotic neighbors on seedling survival and almost never be seen in common methods.

We examined the effect and relative importance of potential explanatory variables by model-average estimator and the sum of the weights for each potential explanatory variable respectively^55^. Firstly, we selected the *m* (from 0 to *n*) explanatory variables from the *n* potential explanatory variables without repeated sampling and combined them as the fixed part of a GLMM, resulting 2^*n*^ (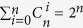) different GLMMs. Secondly, we calculated the weight of each GLMM (equations S1 and S2) and estimated the relative importance of each potential explanatory variable (equations S3 and S4). Finally, we selected the models whose Δ*AIC*_*c*_ ≤ 2 as the optimal model groups, and calculated model-average estimator (equations S5, S6, S7 and S8) of each potential explanatory variable^55^. This process for selecting the models could better reduce the influence of multiple-collinearity among potential explanatory variables in regression models and take into account of all influencing factors comprehensively.

To understand the results of two types of GLMMs, we estimated the adjusted R-squared to evaluate how much the conspecific adult neighborhood indices could explain the distribution of conspecific leaf litter. The response and explanatory variables were sum weight of conspecific leaf litter from October 2011 to October 2013 in each litter trap and the conspecific adult neighborhood indices within 10 m around the focal litter trap respectively.

All of the explanatory variables were subtracted mean of the variable and divided by their own standard deviation (zero-mean normalization). The mean, median and range of the whole potential explanatory variables showed in the Table S4 in appendix. All analyses were conducted in R 3.2.1 (R Development Core Team 2015). The GLMMs were fitted by the “glmer” function of ‘lme4 1.1-9′ package^56^.

## Additional Information

**How to cite this article**: Liu, H. *et al*. Conspecific Leaf Litter-Mediated Effect of Conspecific Adult Neighborhood on Early-Stage Seedling Survival in A Subtropical Forest. *Sci. Rep*. **6**, 37830; doi: 10.1038/srep37830 (2016).

**Publisher's note:** Springer Nature remains neutral with regard to jurisdictional claims in published maps and institutional affiliations.

## Supplementary Material

Supplementary Information
