# Supplementary material for: Conspecific Leaf Litter-Mediated Effect of Conspecific Adult Neighborhood on Early-Stage Seedling Survival in A Subtropical Forest
Source: Sci Rep. 2016 Nov 25;6:37830. doi: 10.1038/srep37830 (PMC5122888; doi:10.1038/srep37830)
Supplement: Supplementary Information [file srep37830-s1.pdf]

Appendix

Title: Conspecific Leaf Litter-Mediated Effect of Conspecific Adult Neighborhood on Early-stage Seedling Survival in A Subtropical Forest

Heming Liu[1,2], Guochun Shen[1,2], Zunping Ma[1,2], Qingsong Yang[1,2], Jianyang Xia[1,2], Xiaofeng Fang[1,2], Xihua Wang[1,2*]

[1]*School of Ecological and Environmental Sciences, East China Normal University, Shanghai 200241, China (Heming Liu: hemingliu1021@gmail.com; Guochun Shen: gcshen@des.ecnu.edu.cn; Zunping Ma: zunpingpier@sina.com; Qingsong Yang: qsyang@des.ecnu.edu.cn; Jianyang Xia: jyxia@des.ecnu.edu.cn; Xiaofeng Fang: fangxiaofeng@gmail.com)*

[2] *Tiantong National Forest Ecosystem Observation and Research Station, Ningbo, Zhejiang 315114, China*

[*] *Correspondence author*:

Xihua Wang, School of Ecological and Environmental Sciences, East China Normal University, NO. 500, Dongchuan Road, Shanghai 200241, China, E-mail: xhwang@des.ecnu.edu.cn

Figure S1 The relative importance of potential factors influencing on early-stage seedlings survival of four species. The potential factors are the log-transformed initial height (HT) of seedlings, conspecific adult neighborhood indices (Acon) and heterospecific (Ahet), the amount of conspecific (Lcon) and heterospecific (Lhet) leaf litter, the density of conspecific (Scon) and heterospecific (Shet) seedling neighbors, canopy openness (Openness), slope (Slope), aspect (transformed by $\cos(\alpha)+1.1$) (Aspect), the elevation (Elevation), pH (pH), total nitrogen (TN), and total phosphorus (TP) in the soil surrounding focal seedling plot. Blue bins indicate significant, positive effects of the variables on seedling survival (The model-average estimator ($\overline{\hat{\beta}}$) > 0); While yellow bins indicate significant, negative effects ($\overline{\hat{\beta}}$ < 0); Gray bins indicate non-significant effects ($\overline{\hat{\beta}}$ = 0). The red and brown arrows highlight the variable of conspecific leaf litter and conspecific adult neighborhood indices, respectively.

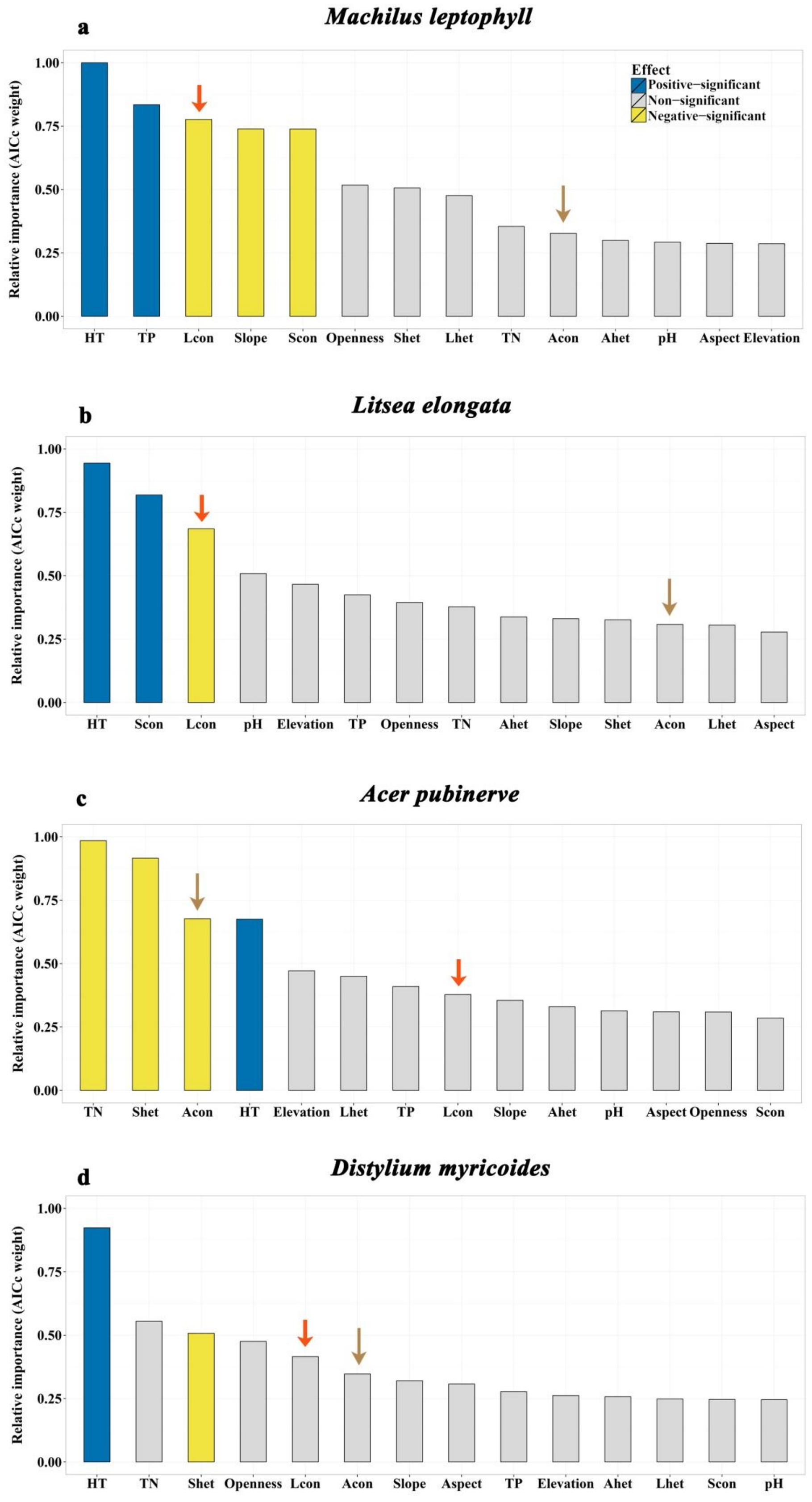
a
Machilus leptophyll
Relative importance (AICc weight)
1.00
0.75
0.50
0.25
0.00
Effect
Positive−significant
Non−significant
Negative−significant
HT TP Lcon Slope Scon Openness Shet Lhet TN Acon Ahet pH Aspect Elevation
b
Litsea elongata
Relative importance (AICc weight)
HT Scon Lcon pH Elevation TP Openness TN Ahet Slope Shet Acon Lhet Aspect
c
Acer pubinerve
Relative importance (AICc weight)
TN Shet Acon HT Elevation Lhet TP Lcon Slope Ahet pH Aspect Openness Scon
d
Distylium myricoides
Relative importance (AICc weight)
HT TN Shet Openness Lcon Acon Slope Aspect TP Elevation Ahet Lhet Scon pH

Figure S2 Relationship between sum of the weight of conspecific leaf litter from 2011 to 2013 in the litter trap and the conspecific adult neighborhood indices of 20 dominant species, i.e, sum of the basal area (m^2) divided by distance (m) from the focal litter trap within 10m is the adjusted R-squared.

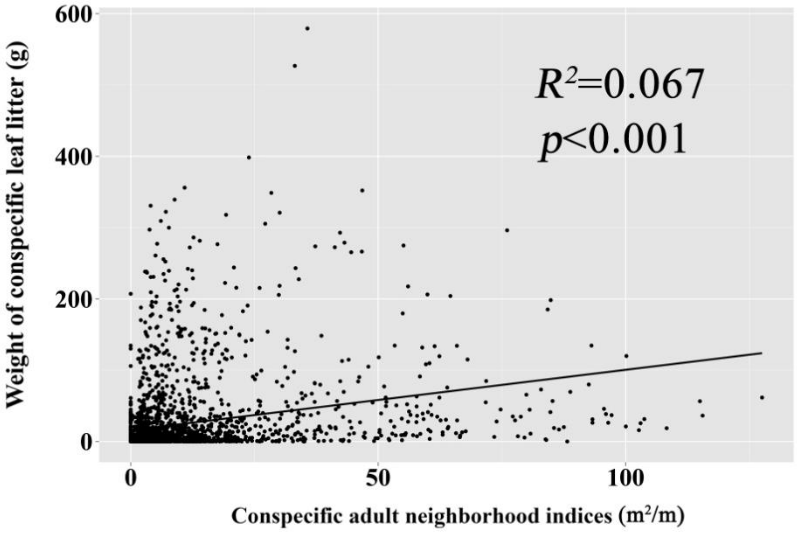

Table S1 The optimal model groups of four species (include the conspecific and heterospecific leaf litter)

Species	Model	AIC_c weight
Machilus leptophylla	$a + b \times \mathrm{HT} + c_1 \times \mathrm{Lcon} + d_1 \times \mathrm{Scon} + c_2 \times \mathrm{Lhet}$	0.1664
	$a + b \times \mathrm{HT} + c_1 \times \mathrm{Lcon} + d_1 \times \mathrm{Scon} + c_2 \times \mathrm{Lhet} + d_2 \times \mathrm{Shet}$	0.1386
	$a + b \times \mathrm{HT} + c_1 \times \mathrm{Lcon} + d_1 \times \mathrm{Scon} + c_2 \times \mathrm{Lhet} + d_2 \times \mathrm{Shet} + e_2 \times \mathrm{Ahet}$	0.1086
	$a + b \times \mathrm{HT} + d_1 \times \mathrm{Scon}$	0.0829
	$a + b \times \mathrm{HT} + c_1 \times \mathrm{Lcon} + d_1 \times \mathrm{Scon} + c_2 \times \mathrm{Lhet} + e_2 \times \mathrm{Ahet}$	0.0822
	$a + b \times \mathrm{HT} + c_1 \times \mathrm{Lcon} + c_2 \times \mathrm{Lhet} + d_2 \times \mathrm{Shet}$	0.0788
	$a + b \times \mathrm{HT} + c_1 \times \mathrm{Lcon} + d_1 \times \mathrm{Scon}$	0.0757
	$a + b \times \mathrm{HT} + c_1 \times \mathrm{Lcon} + c_2 \times \mathrm{Lhet}$	0.0743
	$a + b \times \mathrm{HT} + d_1 \times \mathrm{Scon} + d_2 \times \mathrm{Shet}$	0.0656
	$a + b \times \mathrm{HT} + c_1 \times \mathrm{Lcon} + c_2 \times \mathrm{Lhet} + d_2 \times \mathrm{Shet} + e_2 \times \mathrm{Ahet}$	0.0655
	$a + b \times \mathrm{HT} + c_1 \times \mathrm{Lcon} + d_1 \times \mathrm{Scon} + d_2 \times \mathrm{Shet} + e_2 \times \mathrm{Ahet}$	0.0616
Litsea elongata	$a + b \times \mathrm{HT} + c_1 \times \mathrm{Lcon} + d_1 \times \mathrm{Scon}$	0.4380
	$a + b \times \mathrm{HT} + c_1 \times \mathrm{Lcon} + d_1 \times \mathrm{Scon} + e_2 \times \mathrm{Ahet}$	0.2147
	$a + b \times \mathrm{HT} + c_1 \times \mathrm{Lcon}$	0.1826
	$a + b \times \mathrm{HT} + c_1 \times \mathrm{Lcon} + d_1 \times \mathrm{Scon} + d_2 \times \mathrm{Shet}$	0.1647
Acer pubinerve	$a + d_2 \times \mathrm{Shet} + c_1 \times \mathrm{Lcon}$	0.1644
	$a + d_2 \times \mathrm{Shet}$	0.1352
	$a + d_2 \times \mathrm{Shet} + c_1 \times \mathrm{Lcon} + d_1 \times \mathrm{Scon}$	0.1287
	$a + d_2 \times \mathrm{Shet} + e_2 \times \mathrm{Ahet}$	0.0997
	$a + d_2 \times \mathrm{Shet} + c_1 \times \mathrm{Lcon} + b \times \mathrm{HT}$	0.0846
	$a + d_2 \times \mathrm{Shet} + b \times \mathrm{HT}$	0.0831

	$a + d_2 \times$ Shet + $e_2 \times$ Ahet + $b \times$ HT	0.0795
	$a + d_2 \times$ Shet + $c_1 \times$ Lcon + $d_1 \times$ Scon + $b \times$ HT	0.0774
	$a + d_2 \times$ Shet + $c_1 \times$ Lcon + $e_2 \times$ Ahet	0.0772
	$a + d_2 \times$ Shet + $d_1 \times$ Scon	0.0701
Distylium myricoides	$a + b \times$ HT + $d_2 \times$ Shet	0.2124
	$a + b \times$ HT + $e_1 \times$ Acon + $c_1 \times$ Lcon	0.1498
	$a + b \times$ HT + $e_2 \times$ Ahet	0.0984
	$a + b \times$ HT + $d_2 \times$ Shet + $e_2 \times$ Ahet	0.0965
	$a + b \times$ HT + $d_2 \times$ Shet + $e_1 \times$ Acon	0.0933
	$a + b \times$ HT	0.0917
	$a + b \times$ HT + $d_2 \times$ Shet + $e_1 \times$ Acon + $c_1 \times$ Lcon	0.0884
	$a + b \times$ HT + $e_1 \times$ Acon	0.0865
	$a + b \times$ HT + $d_2 \times$ Shet+ $c_1 \times$ Lcon	0.0830

*Notes:*The independent variables were the log-transformed initial height (HT) of seedlings, the amount of conspecific (Lcon) and heterospecific (Lhet) leaf litter, conspecific adult neighborhood indices (Acon) and heterspecifics (Ahet), the density of conspecific (Scon), and heterospecific (Shet) seedling neighbors.

Table S2 Micro-environment factors of seedling plots

Micro-environment factors	Max value	Min value	Mean value	Standard deviation
Canopy openness(%)	35.31	2.58	9.09	3.86
Elevation(m)	572.28	321.89	441.87	53.9
Slope(°)	82.65	4	35.08	11.98
Aspect(°)	318.92	30	185.93	54.86
TN(g/kg)	10.04	0.95	3.15	1.27
TP(g/kg)	0.79	0.02	0.26	0.13
pH value	5.26	3.5	4.14	0.25

*Notes:*Aspect is the clockwise rotation from the north.

Table S3 The Nagelkerke's R_N^2 of biotic factors and environmental factors among four species

Species	Biotic variables	Environmental variables
Machilus leptophylla	0.1512	0.0462
Litsea elongate	0.3808	0.1397
Acer pubinerve	0.3439	0.4719
Distylium myricoides	0.2739	0.2893

Notes: R_N^2 is calculate by the "r.squaredGLMM" function of ' MuMIn' package; Biotic variables include initial height (**HT**) of seedlings, the amount of conspecific (**Lcon**) and heterospecific (**Lhet**) leaf litter, conspecific adult neighborhood indices (**Acon**) and heterspecifics (**Ahet**), the density of conspecific (**Scon**) and heterospecific (**Shet**) seedling neighbors. Environmental variables include canopy openness, elevation, slope, aspect, pH value, total nitrogen and total phosphorus in the soil.

Table S4 The potential explanatory variables for seedling survival of four species

Variables	Species	Range	Mean	Median
Initial.height(cm)	*Machilus leptophylla*	2.4-32.2	9.8	9.8
	Litsea elongata	3-30.8	7.9	7.2
	Acer pubinerve	2.8-39.5	8.5	7.2
	Distylium myricoides	1-10.4	4.6	4.5
Amount of leaf litter				
Conspecific(g)	*Machilus leptophylla*	0.00-192.08	115.27	137.27
	Litsea elongata	0.00-150.17	29.32	18.3
	Acer pubinerve	0.00-70.84	26.65	15.18
	Distylium myricoides	0.05-149.81	54.42	75.95
Heterospecific(g)	*Machilus leptophylla*	79.72-421.30	151.73	105.95
	Litsea elongata	112.24-389.68	230.69	235.12
	Acer pubinerve	93.24-365.08	180.76	168.06
	Distylium myricoides	67.55-309.98	143.31	114.95
Seedling neighborhood indices (The density of seedlings in seedling plot -1m^2 where was the focal seedling in)				
Conspecific	*Machilus leptophylla*	1-456	209	205
	Litsea elongata	1-26	4	3
	Acer pubinerve	1-23	10	8
	Distylium myricoides	1-15	9	11
Heterospecific	*Machilus leptophylla*	0-31	4	2
	Litsea elongata	0-32	5	4

	Acer pubinerve	0-18	5	3
	Distylium myricoides	0-14	5	4
Adult neighborhood indices (sum of adult basal area(m^2) divided by distance from the focal seedling within 10m)				
Conspecific ($m^2 \cdot m^{-1}$)	*Machilus leptophylla*	0-0.2543	0.1165	0.0872
	Litsea elongata	0-0.0889	0.0228	0.0209
	Acer pubinerve	0-0.1507	0.0245	0.0090
	Distylium myricoides	0.0002-0.0782	0.0345	0.0370
Heterospecific ($m^2 \cdot m^{-1}$)	*Machilus leptophylla*	0.0298-0.4175	0.1119	0.0936
	Litsea elongata	0.0779-0.4641	0.1907	0.1827
	Acer pubinerve	0.0570.-0.2725	0.1703	0.1944
	Distylium myricoides	0.1123-0.2952	0.1876	0.1900

Table S5 Relationship between the conspecific adult neighborhood indices and the dry weights of conspecific leaf litter of the top 20 dominant species in the 20 ha Tiantong dynamic plot.

Species	Adjusted R-squared	p-value
Eurya loquaiana	0.4442	<0.001
Litsea elongata	0.3516	<0.001
Choerospondias axiliaris	0.3469	<0.001
Distylium myricoides	0.4152	<0.001
Lithocarpus henryi	0.4231	<0.001
Cyclobalanopsis sessilifolia	0.2445	<0.001
Camellia fraterna	0.1665	<0.001
Schima superba	0.4993	<0.001
Castanopsis fargesii	0.4842	<0.001
Machilus thunbergii	0.1884	<0.001
Neolitsea aurata var.chekiangensis	0.2359	<0.001
Cleyera japonica	0.0790	0.001
Symplocos anomala	0.0390	0.042
Rhododendron ovatum	0.2811	<0.001
Carpinus viminea	0.1761	<0.001
Castanopsis carlesii	0.2497	<0.001
Machilus leptophylla	0.5990	<0.001
Liquidambar formosana	0.4596	<0.001
Symplocos setchuensis	0.0851	0.013
Acer pubinerve	0.2569	<0.001

Equation S1-S8 How to calculate the Akaike weight of the predictor variable and model-averaged estimator $\bar{\hat{\beta}}$

$$\Delta AIC_{ci} = AIC_{ci} - AIC_{c\min} \tag{S1}$$

$$w_i = \frac{\exp(-\frac{1}{2}\Delta AIC_{ci})}{\sum_{r=1}^{R}\exp(-\frac{1}{2}\Delta AIC_{cr})} \tag{S2}$$

$$I_j(g_i) \begin{cases} 1 & \text{if potential explanatory variable } x_j \text{ is in model } g_i \\ 0 & \text{otherwise} \end{cases} \tag{S3}$$

$$w_+(j) = \sum_{i=1}^{R} w_i I_j(g_i) \tag{S4}$$

$$w_i^{'} = \frac{\exp(-\frac{1}{2}\Delta AIC_{ci})}{\sum_{r=1}^{R1}\exp(-\frac{1}{2}\Delta AIC_{cr})} \tag{S5}$$

$$w_+^{'}(j) = \sum_{i=1}^{R1} w_i I_j(g_i) \tag{S6}$$

$$\bar{\hat{\beta}}_j = \frac{\sum_{i=1}^{R1} w_i^{'} I_j(g_i)\hat{\beta}_{j,i}}{w_+^{'}(j)} \tag{S7}$$

$$\bar{\tilde{\beta}}_j = \sum_{i=1}^{R1} w_i^{'} I_j(g_i)\hat{\beta}_{ji} \tag{S8}$$

Here, AIC_{ci} is the AIC_c of the focal model *i* and $AIC_{c\min}$ is the minimum value of AIC_c in the all *R* models. w_i represents Akaike weight of the focal model *i*. $w_+(j)$ is the sum of Akaike weights through all *R* models where the potential explanatory

variable *j* appears in the model and denotes the relative importance of the potential explanatory variable *j*. Note, $w_i^{'}$ and $w_+^{'}(j)$ represent Akaike weight of the focal optimal model *i* and explanatory variable *j* in the *R1* optimal models. $\hat{\beta}_{j,i}$ denotes the estimator of β_j based on the optimal model g_i (If β_j is no significant difference with 0 or potential explanatory variable *j* does not appear in the optimal model g_i , the $\hat{\beta}_{j,i}$ and $I_j(g_i)$ both are 0). $\hat{\bar{\beta}}_j$ is a model-averaged estimator of variable *j* in the optimal model groups. Note, if $\hat{\beta}_{j,i} \equiv 0$, then the second model-averaged estimator ($\tilde{\bar{\beta}}_j$) will be instead of model-averaged estimator ($\hat{\bar{\beta}}_j$).
